# Cell Population Dynamics in Wound-Induced Hair Follicle Neogenesis Model

**DOI:** 10.3390/life12071058

**Published:** 2022-07-15

**Authors:** Maria Helm, Juliane Loui, Jan C. Simon, Ruben A. Ferrer

**Affiliations:** Department of Dermatology, Venerology and Allergology, Leipzig University Medical Center, Philipp-Rosenthal-Straße 23, 04103 Leipzig, Germany; maria.helm@medizin.uni-leipzig.de (M.H.); juliane.loui@medizin.uni-leipzig.de (J.L.); jan-christoph.simon@medizin.uni-leipzig.de (J.C.S.)

**Keywords:** wound-induced hair follicle neogenesis, flow cytometry, regeneration

## Abstract

Hair follicle (HF) regeneration can be achieved in the center of large full-thickness wounds on mouse backs (wound-induced HF neogenesis model, WIHN). Investigations with this model have allowed for the identification of some of the factors limiting the extent of fibrosis, which creates a permissive environment for the reposition of HF. For WIHN, specific subpopulations of cells rather than cell types are permissive to this process. Detailed information on the cellular composition in WIHN is not available. Here, we provide a description of changes in cell numbers of fibroblasts, HF dermal papilla, endothelial cells, keratinocytes (interfollicular epidermis, HF-infundibulum, HF-isthmus, HF-bulge (basal and suprabasal), HF-hair germ) and immune cells (macrophages, monocytes, dendritic cells, T cells (CD4^+^, CD8^+^, CD4^+^/CD8^+^, regulatory T cells) and neutrophils) based on flow cytometric analysis. We compared unwounded skin with large wounds (1.5 × 1.5 cm) at different time points after wounding. We found that non-immune dermal cells have the largest share in the skin at all time points studied, and that the number of epidermal cells started increasing nine days after wounding, which precede isthmus cells and bulge cells, mirroring the development of hair follicles. Monocytes and neutrophils represent most myeloid cells in wounds and remain in wounds even beyond the inflammatory phase of wound healing. Macrophages can be identified as inflammatory and alternative cells and are also found in wounds even in the late remodeling phase of wound healing. Lastly, we provide information about T cells in large wounds. Most T cells in the wounds were CD8^+^ at all time points and expressed γδTCR, which was previously thought to be expressed mainly on CD4^+^. We also report the existence of double positive CD4/CD8. Our study provides a guide in terms of time points suitable for the further study of cell subpopulations aiming to dissect the cellular heterogeneity in WIHN. Our results might set the base for the comparison of WIHN between control mice and animals manipulated to influence HF neogenesis and the full understanding of the responsible actors allowing for HF regeneration.

## 1. Introduction

The wound-induced hair follicle (HF) neogenesis model (WIHN) allows the studying of the processes responsible for communication between fibroblasts (fib), immune cells and keratinocytes resulting in restoration of lost HF upon injury [1]. This model is performed by generating large wounds (at least 1 cm^2^) on the backs of adult mice. Different to smaller wounds, these large wounds are able to regenerate HF in their centers [1,2]. A central question in this model deals with how the same cells and pathways orchestrate fibrosis/scar formation or regeneration of HF, two opposing fates within a wound [3]. The fact that the fib activation of canonical Wnt signaling is central for WHIN and for scarring perfectly exemplifies this issue [1,2,4,5]. Another example is the involvement of “alternatively activated” macrophages (Mac) in both processes [3]. It seems specific cell subpopulations rather than cell types are either permissive to HF regeneration or conducive to scarring [6]. This calls for further studies dissecting the complexity of these cell subpopulations, including rare or low-frequency cells.

Methods such as cell sorting for isolating subpopulations for further assays require previous knowledge of markers. Newer methods such as single-cell RNAseq or cytometry by time of flight (CyTOF) can potentially overcome this limitation. In this regard, these methods have been used recently to characterize the cellular composition of large WIHN wounds [6,7,8]. However, many investigations have focused on time points occurring after full re-epithelialization has occurred (after post-wounding day (pw) 12). Nevertheless, it is known that events occurring before this time point prime the wounds for HF neogenesis [3,6,9]. These events are orchestrated by inflammatory cells interacting with different epidermal and stromal cell subpopulations. However, knowledge regarding the proportion and type of immune cells infiltrating into the wounds before pw12 and egressing them after this time point is not available. The same is true for stromal cells and epidermal cells. Because of this, an estimation of which time points should be further analyzed before and after pw12 is difficult. Knowing beforehand the relevant time points for analysis increases the chance of finding even the rarest of low-frequency cell subpopulations. While the cell composition and their time dynamics have been better characterized in small murine wounds, this information is not available for large WIHN wounds.

Here, we analyze changes in the cell numbers of dermal cells (fib, DP = dermal papilla, endothel, IFE), keratinocytes (interfollicular IFE, infundibulum (Inf), isthmus (Ist), bulge (Bul, basal and suprabasal), hair germ (HG)), immune cell populations (mac), monocytes (mono), dendritic cells (DC), T cells and neutrophils based on flow cytometric analysis, which can be used as a guide for future studies. Our study can set the base for further investigations aiming to detangle the complex dynamics of cells in WIHN. This knowledge could be exploited to define targets for manipulation in wounds aiming to decrease scar formation and improve regeneration of skin appendages after trauma.

## 2. Materials and Methods

### 2.1. Experimental Animals

Male and female wild-type C57BL/6N mice were used for this study. All experiments were approved by the Animal Welfare Committee of the Saxonian Government (TVV 31/20). Animals were anesthetized using a Ketamine/Rompun mixture (0.25 mg/2.5 mg per mouse) through intraperitoneal (i.p.) injection.

### 2.2. Wounding Model

Full-thickness square wounds (1.5 × 1.5 cm^2^) were performed on the back after disinfection of the area with 70% ethanol as previously described [2]. The wounds were left undressed. Operative and postoperative pain was prevented by supplementation of drinking water with Tramadol (150 µg/mL) ad libitum 24 h before surgery and after wounding until sacrifice of the animals.

### 2.3. Skin Digestion and Preparation of Single-Cell Suspension for Flow Cytometry

Unwounded skin (pw0), skin after 12 h (pw12h) or skin at day after wounding (pw) 1, 3, 5, 7, 9, 11, 13, 15, 17 and 23 was used for analysis. At these time points, mice were euthanized through cervical dislocation and heart puncture after i.p. anesthesia with Ketamine/Rompun. The unwounded back or the wound site was shaved with clippers and disinfected with 70% ethanol. The wounds were dissected including 1–2 mm of wound margin without the panniculus carnosus. The wounds were minced into small pieces (1–2 mm) and incubated in digestion media (RPMI with 100 U/mL Pen/Strep and 1.7 U/mL Liberase^TM^ DL from Sigma, Roche Diagnostics GmbH, Mannheim, Germany) in a 37 °C water bath for 30 min with vortexing every 5 min. To stop the digestion, RPMI supplemented with 10% FBS was added. The suspension was further homogenized using serological pipettes. The cell suspension was filtered through 100 µm, 70 µm and 40 µm mesh with washing steps using PBS + 5% FBS. At least 3 animals (male and female) were used to generate the data at each time point.

### 2.4. Flow Cytometry Staining

The single-cell suspension was stained with different antibodies against cell surface markers and isotype controls in flow cytometry buffer (PBS supplemented with 5 % FBS, each 1:100). A list of the antibodies used for this study can be found on Table 1. The cells were stained at 4 °C for 25 min in the dark. Afterwards, either Zombie NIR (1:50) or propidium iodine (1:100) was added and incubated for a further 5 min at 4 °C. Then, the cells were spun down at 8050 RCF× *g* at 4 °C for 7 min and washed twice with flow cytometry buffer and centrifuged. The cells were then resuspended and transferred to FACS tubes. For intracellular staining of FOXP3, the cells were transferred into 15 mL tubes and treated with True-Nuclear™ Transcription Factor kit from BioLegend^®^ (Cat.: 424401, BioLegend, San Diego, CA, USA) and stained with either an antibody against FOXP3 or an isotype control following the manufacturer’s instructions.

### 2.5. Acquisition of Flow Cytometry Data and Analysis

The stained cells were analyzed on a BD FACS Canto^TM^ II cytometer with BD Diva Software (Version 9.0.1, Firmware Version: 1.87 (BD FACSCantoII). We recorded at least 10,000 events per tube. Gates for expression of the different markers were set according to unstained or isotype controls. Dot plots and histograms generated with BD Diva software were stored as PDF files and used to generate Excel/Graph Pad tables and graphs.

### 2.6. Statistics

Experiments were conducted with at least three wounds each from one mouse (female or male). We included at least 3 mice per time point and group. Where required, the means of two post-wounding days (pw) were compared using a *t*-test. The results are expressed in graphs as mean ± SEM.

## 3. Results

**Figure 1:**
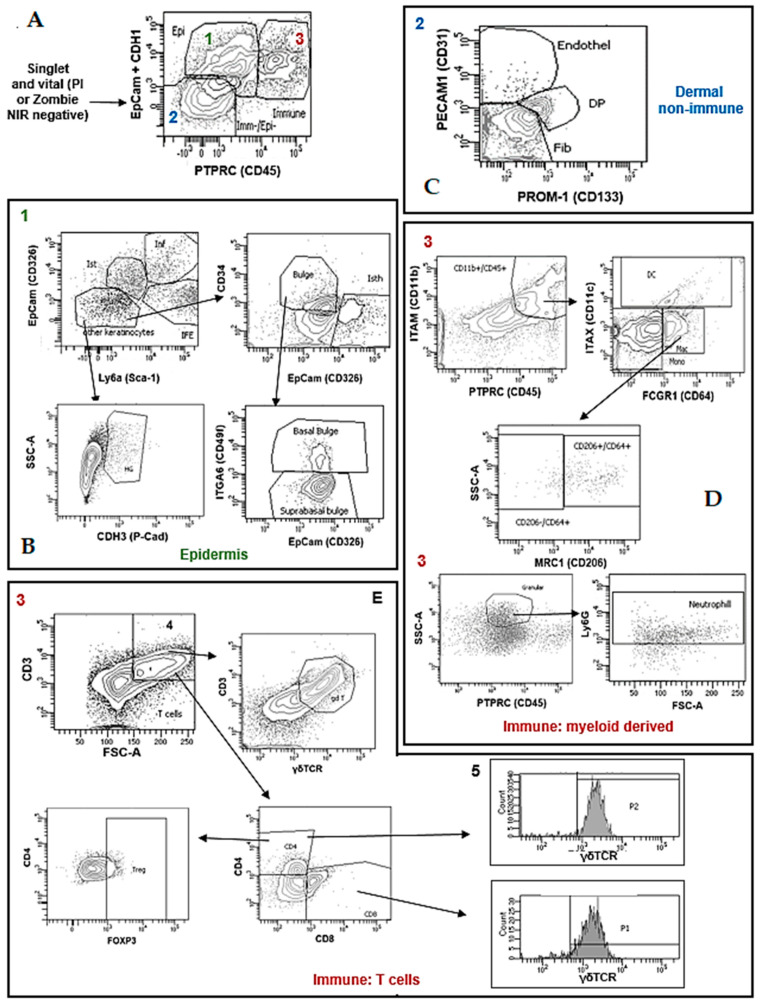
**Gating Strategy for Defining Cell Populations.** Dot plots show representative data from pw0 unwounded skin, which represented our control situation. (**A**) The analysis using flow cytometry started by including negative singlet events for dead cell markers such as PI or Zombie NIR (so-called vital cells). Singlet and vital cells were divided into 3 compartments (1: CDH1(CD324)^+^/EpCam(CD326)^+^ Epithelial (Epi); 2: CDH1^−^/EpCam^−^/PTPRC(CD45)^−^ dermal non-immune cells (Imm-/Epi-); 3: PTPRC^+^ immune cells (Immune)). (**B**) Epithelial cells were further divided into IFE, Inf, Ist, Bul and HG using the markers Ly6A, EpCam, CD34, CDH3(P-Cam) and ITGA6(Integrin alpha 6). (**C**) Dermal non-immune cells were classified as PECAM1(CD31)^+^ endothelial cells (endothel), PROM-1(CD133)^+^ DP, or Fib (PECAM1^−^/PROM-1^−^). Immune cells were further divided into myeloid derived cells and T cells. (**D**) Myeloid derived cells expressing ITAM(CD11b) were subdivided into ITAX(CD11c)^+^ DC, ITAX^−^/FCGR1(CD64)^−^ mono, and ITAX^−^/FCGR1^+^ mac. Mac cells were classified into MRC1(CD206) ^+^ or ^−^ cells. Highly granular (SSC-A high) PTPRC^+^ cells in myeloid derived compartments were also analyzed regarding Ly6G expression to define neutrophils. (**E**) T cells in immune compartments were defined as CD3^+^ cells and further divided regarding CD4, CD8 and δγTCR expression, while CD4^+^ cells were also analyzed regarding expression of FOXP3 to identify Treg.

We employed flow cytometry (FC) to study the composition of large wounds (1.5 × 1.5 cm) on the backs of wild-type C57BL/6N mice. These populations were defined by adapting a combination of markers taken during previous investigations into unwounded skin and wounds [8,10,11,12]. The gating strategy used for defining the populations is depicted in Figure 1. A list of the staining panels used for defining the populations can be found in Table 2. This combination of markers allows for the identification of several cell populations at different time points during WIHN, which is currently not available.

### 3.1. Changes in Composition of the Skin/Wounds

**Figure 2:**
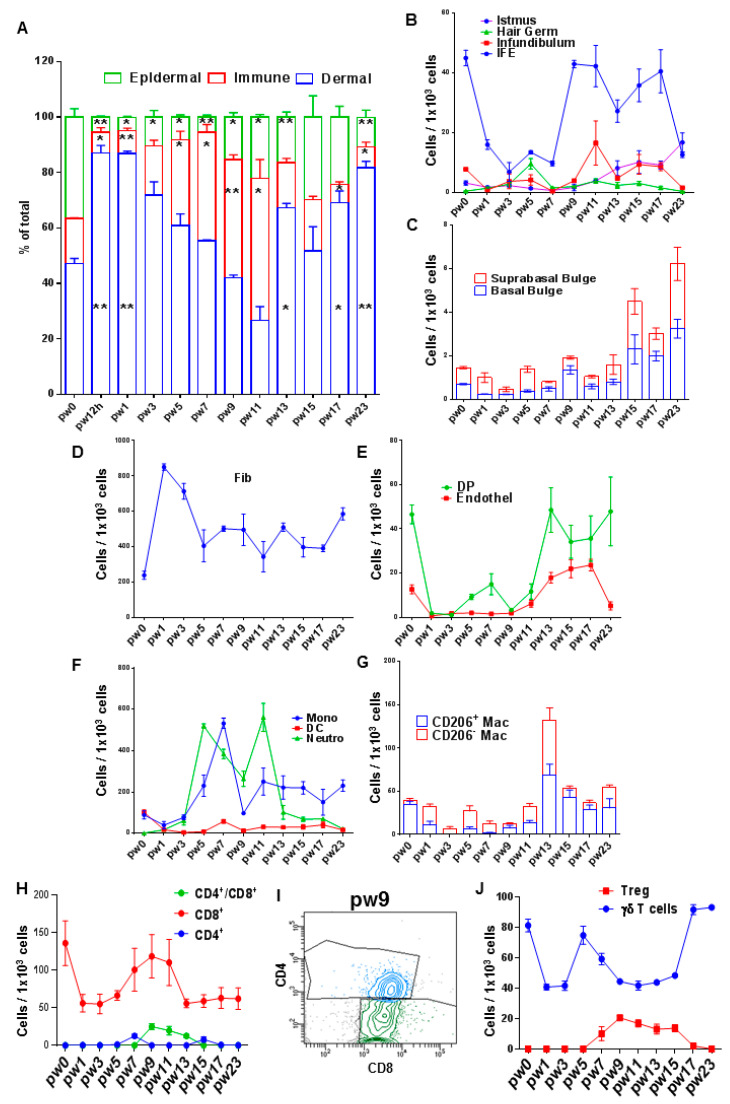
Changes in cell numbers for cell populations in unwounded skin and WIHN wounds. Cells were isolated from digested unwounded skin (pw0) or at different pw along wound healing, stained with antibodies against cell surface markers or intracellular FOXP3 and analyzed with flow cytometry. (**A**) Composition of skin (pw0) or wounds (other pw) in terms of dermal compartment, immune cells and epidermis. (**B**) Changes in epithelial cell number expressed as cell/1 × 10^3^ CHD1^+^ cells. (**C**) Changes in suprabasal and basal Bul. (**D**) Changes in numbers for fib expressed as cell/1 × 10^3^ CHD1^−^/PTPRC^−^ cells. (**E**) Changes in DP and endothelial cells expressed as cell/1 × 10^3^ CHD1^−^/PTPRC^−^ cells. (**F**) Changes in numbers for DC, mono and neutrophil expressed as cell/1 × 10^3^ PTPRC^+^ cells. (**G**) Changes in CD206(MRC1) ^+^ and ^−^ cells expressed as cell/1 × 10^3^ PTPRC^+^ cells. (**H**) Changes in numbers for T cells expressed as cell/1 × 10^3^ PTPRC^+^/CD3^+^ cells. (**I**) Exemplary dot plot of CD4 and CD8 expression of CD3^+^/PTPRC^+^ cells at pw9. (**J**) Changes in numbers for δγT cells expressed as cell/1 × 10^3^ PTPRC^+^/CD3^+^ cells and Treg expressed as cell/1 × 10^3^ PTPRC^+^/CD3^+^/CD4^+^ cells. Results are expressed as mean ± SEM, *n* = 3–8. Comparison between pw0 and subsequent pw values was carried out individually with a *t*-Test, *p* value = * ≤ 0.05, ** ≤ 0.01.

We analyzed the proportion of epithelial cells, dermal cells and immune cells as defined in gates 1, 2 and 3 in Figure 1. Dermal cells have the largest share in cell composition in unwounded skin and at all pw values (Figure 2B). Epithelial cells start expanding from pw9 onwards, while immune cells reduce their share in wounds compared to unwounded skin during pw12h and pw1 and start expanding at pw3 until pw13 (Figure 2A). To our knowledge, these results have not been reported before for this model. To analyze the difference between the numbers of each population comparing the different time points studied here, we used the *t*-Test, since we did not know the expected frequencies for this model.

### 3.2. Changes in Epithelial Compartment

Most epithelial cells in unwounded skin and at all pw values were IFE keratinocytes (Figure 2B). Information regarding the changes in the numbers of HF keratinocytes along the whole course of wound healing in large wounds was not previously available and is now provided in our study. The HG compartment showed an expansion at pw5 and at pw 11 (Figure 2B). Inf cells increased their numbers at pw11 preceding Ist cells at pw13, while bulge cells were the last to augment their numbers, peaking at pw13 and onwards (Figure 2B,C).

### 3.3. Changes in Dermal Compartment

Fib numbers peaked at pw1 and then decreased within the dermal fraction (Figure 2D). Afterwards, fib progressively decreased from pw7 and onwards (Figure 2D). DP cells represented less than 5% of dermal cells and their numbers increased at pw7 and from pw13 onwards (Figure 2E). Endothelial cells represented an even smaller fraction of dermal cells but showed a similar cell number dynamic to DP (Figure 2E).

### 3.4. Changes in Myeloid Cell Compartment

The analysis of the myeloid derived cell compartment shows that most cells here are represented by neutrophils and monos. Neutrophils and monos can be readily detected in unwounded skin and increased their numbers in the myeloid fraction from pw3 and onwards (Figure 2F). Both cell populations presented a dip in their numbers at pw9, which coincided with an expansion of keratinocytes in the wounds (Figure 2A,F). Neutrophils started disappearing from the wounds at pw13, while monos started decreasing at pw11 butwere still present at pw23 (Figure 2F). DC also changed their contribution to the myeloid cell compartment over the course of WIHN healing (Figure 2F). In unwounded skin, they showed a similar number to mono (Figure 2F). During wound healing, DC peaked again at pw7 and remained at a lower level compared to pw0 until pw23 (Figure 2F). The majority of the mac cells in pw0 skin were MRC1/CD206^int/high^, which corresponds to resident mac (Figure 1). During wound healing, MRC1/CD206^−^ (inflammatory mac) reached a peak (together with MEC1/CD206^+^ mac) at pw13 (Figure 2G).

### 3.5. Changes in T Cell Compartment

Analysis of the T cell populations showed that most of these cells were CD8^+^ and that T cells (both CD8^+^ and CD4^+^) increased in wounds from pw5 to pw13 (Figure 2H). CD4^+^ cells represented a small fraction in this compartment and were CD8^+^ at pw9-13 (Figure 2H,I). The existence of these double positive cells has been reported in peripheral blood and in several tissues including skin [13]. γδ T cells are required for WIHN [2]. Almost all CD4^+^ and CD8^+^ T cells were γδTCR^+^ (exemplary histogram in Figure 1). As a cell population on its own (PTPRC^+^/CD3^+^), γδ T cell numbers decreased in skin after wounding, peaked again at pw5, dipped again and then reached the pw0 number by pw23 (Figure 2J). Tregs (FOXP3^+^ cells) among CD3^+^/CD4^+^ appeared in wounds at pw5 and peaked at pw9 (Figure 2J).

## 4. Discussion

This study describes a strategy to analyze multiple populations within large murine wounds, which can regenerate HF in their centers. Although newer methods such as scRNAseq and CyTOF could be used to study cell subpopulations with spectacular detail, such techniques are very expensive. An additional problem for applying these techniques is represented by the fact that many cell types, for example, γδ T cells, are very scarce, and their analysis should include previous knowledge about which time points during wound healing are more suitable for studying them, and whether an enrichment step should be performed before proceeding with analysis. Our data might aid in guiding these steps. We do not aim to provide here a definitive guide on the cell composition of large regenerative wounds, but to set the stage for further investigations which will further dissect the cell heterogeneity in the WIHN model. It is worth mentioning that the data presented here show the changes in the percentage of cells defined as keratinocytes, dermal cells, or immune cells from total living cells, but do not show the actual cell number of each cell type given the variability in this variable depending on the effectivity of skin digestion, the staining conditions for flow cytometry and the interindividual variations in mice. Establishing actual ranges for cell numbers in this model would require studying more animals with the normal values for this mouse strain.

In the case of IFE, our FC strategy is able to show the expected composition of epidermis having more IFE keratinocytes than other keratinocyte populations [6] (Figure 2B). Information regarding changes in the numbers of HF keratinocytes along the whole course of wound healing in large wounds was not previously available and is now provided in our study. The HG compartment presents two time points with number expansion, and it is attractive to hypothesize that perhaps the first time point corresponds to proliferation of cells from HF surrounding the wounds, whereas the later time point could represent proliferation of HG in HF inside wounds. Nevertheless, these time points should be used for further studies, for example, separating the wounds into centers and margins before digestion as well as combining FC studies with, for example, immunofluorescence to determine localization of proliferating HG cells. Interestingly, our study was able to capture a sequential expansion of Inf, Ist and bulge cells, which mirrors HF development [14]. Our study suggests that changes in HF-keratinocyte populations should focus on time points starting at pw5 to increase the chances of detecting subpopulations responsive to pro WIHN stimuli.

We focused our investigation in the dermal, non-immune compartment on Fib, DP and endothelial cells. We observed a dynamic of decreasing Fib contribution to the cell number in whole wounds during the first 7 days after wounding, probably caused by an expansion of immune cells during the inflammatory phase. Both DP and endothelial cells started expanding within the dermal compartment at pw7, which indicates that further studies on dermal cell populations should focus on time points around pw7 and onwards. Information in the literature regarding changes in the number of dermal cells in wounds is very scarce, and there are only data for time points where fibs enter the wound bed and when neogenic vessels sprout within small wounds [15] but not for the WIHN model.

We also characterized immune cells, both myeloid-derived and T cells. As expected for a wound, neutrophils and monos represented most inflammatory cells. These cells peaked later and remained longer in the wounds compared to small wounds [8,16] (Figure 2F). So far, there is no information regarding number of DC in large neogenic wounds. For mac, we focused on non-inflammatory MRC1/CD206^int/high^ and inflammatory MRC1/CD206^−^ cells. Both cell types peaked at pw13 in WIHN wounds compared to small wounds, where mac peaked at early time points during wound healing [8]. Whether this influences the capacity of large wounds to regenerate HF remains to be investigated. Although mac cells are required for wound healing, reducing their numbers in the later time points of the remodeling phase seems to improve WIHN [3]. This raises the attractive hypothesis that by delaying the influx of mac into wounds, the environment favors HF regeneration. Further studies can add to the data presented here, by, for example, including further markers such as CCR2 and CXCR3, among others, to differentiate cells that migrate into the wounds from resident cells and can be combined with immunofluorescence to establish the spatial localization of distinct mac populations in the wounds, which might influence regenerative capacity. Our study might aid in selecting proper time points to further investigate the role of inflammatory and alternative cells. T cells have received the least attention in the WIHN model, although FGF9 secreted by γδ T cells is required for this process [2]. Besides the aforementioned cells, we also provide information regarding CD4^+^ and CD8^+^ T cells, as well as previously unreported CD4^+^/CD8^+^ cells. The existence of these double positive cells has been reported in peripheral blood and in several tissues including skin [13]. The importance and further identity and localization of T cell subpopulations should be further explored using pre-sorting of PTPRC^+^/CD3^+^ cells to enrich for T cells and should prospectively combine the further dis` of FC with other technologies such as scRNAseq, CyTOF and immunofluorescence. Regarding γδ T cells, we show that a substantial percentage of CD4^+^ and CD8^+^ T cells are γδTCR^+^. There is still a lack of clarity in the literature regarding the whole profile of these cells. For WIHN, FC analysis has only been completed after digesting dermis from wounds, while we used whole skin for our analysis, which might explain the discrepancies with the work from [2], which shows an increase in the percentage of dermal cells being γδ T cells starting at pw9 and onwards. Tregs are also required for HF activation and regeneration [17]. We show a peak for these cells at pw9, an important day for WIHN, marking start of Wnt activation in the transition to HF regeneration activation [1,2,18]. To our knowledge, our study provides non-available information regarding changes in T cells along the healing of large wounds, including different T cell subpopulations.

In summary, our study can be used as a guide for the selection of relevant time points to deepen analysis of specific cell types and to understand the changes in cellular composition in WIHN. Our study also sets a base for the comparison of WIHN between control mice and animals manipulated to influence HF neogenesis, and between large and small wounds using other multimodal technologies, for example combining transcriptomics, proteomics, FC and CyTOF together with immunofluorescence.

## Figures and Tables

**Table 1:** List of Antibodies Used in this Study.

Antibody	Manufacturer	Cat-Number	Dilution
Alexa Fluor^®^ 488 anti-mouse FOXP3	BioLegend^®^	126405	1:100
Alexa Fluor^®^ 488 anti-mouse TCR γ/δ	BioLegend^®^	118127	1:100
Anti-mP-Cadherin—APC-conjugated	R&D Systems, Minneapolis, MN, USA	FAB761A	1:100
APC anti-mouse CD11c	BioLegend^®^	117309	1:100
APC anti-mouse CD133	BioLegend^®^	141207	1:100
APC anti-mouse CD206	BioLegend^®^	141708	1:100
APC anti-mouse CD34	BioLegend^®^	128612	1:100
APC anti-mouse CD4	BioLegend^®^	100411	1:100
APC/Cy7 anti-human/mouse CD49f	BioLegend^®^	313627	1:100
Brilliant Violet 421^TM^ anti-mouse CD326 (Ep-CAM)	BioLegend^®^	118225	1:100
Brilliant Violet 421^TM^ anti-mouse CD45	BioLegend^®^	103134	1:100
Brilliant Violet 421^TM^ anti-mouse CD8a	BioLegend^®^	100753	1:100
BV421 Anti-Mouse CD26	BD OptiBuild^TM^ Heidelberg, Germany	740021	1:100
FITC anti-mouse CD326 (Ep-CAM)	BioLegend^®^	118207	1:100
FITC anti-mouse/human CD11b	BioLegend^®^	101205	1:100
FITC Mouse Anti-E-Cadherin	BD Transduction Laboraties^TM^, Amsterdam, The Netherlands	612130	1:100
PE anti-mouse CD3	BioLegend^®^	100205	1:100
PE anti-mouse CD31	BioLegend^®^	102407	1:100
PE anti-mouse CD45	BioLegend^®^	103106	1:100
PE anti-mouse Ly-6G	BioLegend^®^	127607	1:100
PE anti-mouse/human CD324 (E-Cadherin)	BioLegend^®^	147304	1:100
PE/Cyanine7 anti-mouse CD64 (FcγRI)	BioLegend^®^	139313	1:100
PerCP/Cyanine5.5 anti-mouse CD45	BioLegend^®^	103132	1:100
PerCP/Cyanine5.5 anti-mouse Ly-6A/E (Sca-1)	BioLegend^®^	108123	1:100
Propidium iodide	Molecular probes (Thermo Fischer Scientific, Eugene, OR, USA)	P1304MP	1:100
Zombie NIR^TM^ Dye	BioLegend^®^	77184	1:50

**Table 2:** Combination of markers used in this study.

**Epithelial 1**	**Epithelial 2**
Brilliant Violet 421^TM^ anti-mouse CD45	APC anti-mouse CD34
FITC anti-mouse CD326 (Ep-CAM)	Brilliant Violet 421^TM^ anti-mouse CD326 (Ep-CAM)
Anti-mP-Cadherin—APC-conjugated	FITC Mouse Anti-E-Cadherin (CD324)
PerCP/Cyanine5.5 anti-mouse Ly-6A/E (Sca-1)	INGA6 (CD49f) APC-Cy7
PE anti-mouse E-Cadherin (CD324)	PE anti-mouse CD45
	PerCP/Cyanine5.5 anti-mouse Ly-6A/E (Sca-1)
Zombie NIR^TM^ Dye	Propidium iodide
**Macrophages**	**Monocytes/Dendritic Cells**
APC anti-mouse CD206	APC anti-mouse CD11c
FITC anti-mouse/human CD11b	FITC anti-mouse/human CD11b
PE anti-mouse Ly-6G	PE anti-mouse Ly-6G
PE/Cyanine7 anti-mouse CD64 (FCγRI)	PE/Cyanine7 anti-mouse CD64 (FCγRI)
PerCP/Cyanine5.5 anti-mouse CD45	PerCP/Cyanine5.5 anti-mouse CD45
Propidium iodide	Propidium iodide
**T Cell**	**Treg**
Alexa Fluor^®^ 488 anti-mouse TCR γ/δ	Alexa Fluor^®^ 488 anti-mouse FOXP3
APC anti-mouse CD4	APC anti-mouse CD4
Brilliant Violet 421^TM^ anti-mouse CD8a	Brilliant Violet 421^TM^ anti-mouse CD8a
PE anti-mouse CD3	PE anti-mouse CD3
PerCP/Cyanine5.5 anti-mouse CD45	PerCP/Cyanine5.5 anti-mouse CD45
Zombie NIR^TM^ Dye	Zombie NIR^TM^ Dye
**Dermal cell (Fib, DP, Endothel)**	
FITC Mouse Anti-E-Cadherin (CD324)	
APC anti-mouse Prominin (CD133)	
Brilliant Violet 421^TM^ anti-mouse CD326 (Ep-CAM)	
PE anti-mouse CD31	
PerCP/Cyanine5.5 anti-mouse CD45	
Zombie NIR^TM^ Dye	

## Data Availability

The authors can make raw data (Excel tables) as well as flow cytometry histograms/dot plots (PDFs) available upon request.
